# Micro-Scale Numerical Simulation of Fatigue Failure for CFRP Subjected to Multiple-Amplitude Cyclic Loadings Based on Entropy Damage Criterion

**DOI:** 10.3390/ma16186120

**Published:** 2023-09-07

**Authors:** Huachao Deng, Keitaro Toda, Mio Sato, Jun Koyanagi

**Affiliations:** 1 Department of Materials Science and Technology, Tokyo University of Science, Tokyo 125-8585, Japan; 2 Japan Aerospace Exploration Agency, Osawa, Tokyo 181-0015, Japan

**Keywords:** matrix resin failure, entropy-based strength degradation, multiple-amplitude cyclic loadings, finite element simulation

## Abstract

Fatigue failure of carbon fiber-reinforced plastics (CFRPs) under cyclic loadings has attracted the attention of researchers recently. In this study, the entropy-based failure criterion is proposed to investigate the fatigue lifetime of unidirectional CFRPs subjected to multiple-amplitude cyclic loadings. Due to the heterogeneity of CFRPs, a micro-finite element model considering matrix resin and fibers independently is developed, and the entropy-based damage criterion is implemented into a user-subroutine of Abaqus to model the progressive damage of matrix resin. The fatigue lifetime of CFRPs under typical loading sequences consisting of two stages, such as varying from low to high (L-H) or from high to low (H-L) loading sequence, is estimated with the proposed failure criterion. Numerical results show that the initial damage occurs near the area between two fibers, and a transverse crack propagates progressively under the cyclic loading. The difference in predicted lifetime to final failure in L-H and H-L stress levels is 6.3%. Thus, the effect of loading sequence on the fatigue lifetime can be revealed via the proposed entropy-based damage criterion. Comparisons with the conventional linear cumulative damage (LCD) and kinetic crack growth (KCG) theories are also conducted to demonstrate the validity of the proposed method. The entropy-based failure criterion is a promising method to predict the residual strength and fatigue lifetime of CFRP components.

## 1. Introduction

Due to their excellent corrosion resistance, specific strength and modulus, CFRPs are being widely utilized in the aerospace [1,2], automotive [3,4], construction industries [5,6], lightweight structures [7,8] and military fields [9,10]. Differing from conventional materials, CFRPs usually consist of a polymer matrix and fibers [11], in which the polymer matrix usually exhibits visco-elastic behavior [12]. During service periods, CFRPs are subjected to arbitrary and multiple-amplitude loadings, and the failure mechanisms, such as transverse cracks [13,14,15,16] and interface delamination [17], are also time- and temperature-dependent [18]. It should be noted that the loading sequences are usually randomly applied on the CFRP components [19,20,21]. Accurate prediction of fatigue lifetimes is crucial for the integrity of CFRP components [22,23]. However, investigations into the fatigue lifetime of CFRPs under random loadings are still limited. Thus, the development of effective methodologies to estimate the fatigue lifetimes of CFRP components under random loadings [24,25] is of great significance.

In early works, the investigations into lifetime estimations of CFRPs were mainly conducted using the experimental method. Based on the experimental results [26], it was found that fiber-reinforced plastics suffered from fiber breakage, transverse cracks and interface delamination, in which the transverse cracks accounted for a large portion of the total period until final fatigue failure. Ogihara et al. [27] conducted a constant amplitude fatigue test of quasi-isotropic CFRP laminates, and experimental results revealed that the load level and the number of cycles were two significant factors affecting transverse crack accumulation. Li et al. [28] investigated the effects of elevated temperature and hydraulic pressure on the long-term properties of a carbon/glass hybrid rod. In addition to the stress level and number of loading cycles, it was also known that load frequency was a key factor affecting the fatigue performance of polymer–matrix composites. Miyano et al. [29] studied the fatigue strength of CFRP laminates and developed a prediction method to address the effects of load frequency and stress ratio based on certain hypotheses. It is worth noting that under the given loading cycles, a lower loading frequency would result in greater damage than a higher one [30,31]. Additionally, the applications of CFRPs for lightweight structures [32,33,34] are also notable. Chen et al. [35] tested the mechanical performance and investigated the progressive failure characteristics of CFRP/aluminum joints for lightweight applications. Zhang and An [36] proposed a topology optimization of composite structures for additive manufacturing. To analyze the failure behaviors of CFRPs for lightweight vehicle applications, Sun et al. [37] developed an integrated computational materials–engineering framework at the same time.

Recently, micro-scale modeling was also proposed to investigate the failure mechanisms of CFRPs, in which both the polymer matrix and fibers were considered. Asp et al. [38] presented a scheme of critical dilatational deformation in the first quadrant of the bi-axial failure envelope for polymers, which physically suggested that cavitation or crazing occurs in polymer materials. Based on the work of Asp et al. [38], a strain-invariant failure theory and element-failure method were also developed [39,40]. Canal et al. [41] implemented a periodic unit-cell simulation of matrix failure with an elasto-visco-elastic constitutive law. However, the above-mentioned literature only addresses static failure. To address the time-dependence of the matrix, Schapery [12] proposed methods for evaluating the properties of polymeric materials with some specific constitutive equations derived from thermodynamic principles. McCartney [42,43] derived a theory of crack propagation in a linear visco-elastic material, based on an energy balance fracture criterion. Based on the linear cumulative damage law (Miner’s law), Christensen [44,45] developed the kinetic crack growth theory to estimate the lifetime of polymeric materials. Additionally, Koyanagi et al. [46] developed an elasto-visco-plastic constitutive equation using continuum damage mechanics to simulate the strain-rate dependent transition of the transverse tensile failure mode in fiber-reinforced composites. Numerical results showed that in the case of high strain rates, interface failure was the dominant failure mode, while matrix failure was dominant at low strain rates. Nevertheless, the existing methods were not suitable for estimations of the fatigue lifetime and residual strength of CFRPs subjected to randomly loading sequences.

According to thermodynamics, when solid materials are subjected to thermomechanical loadings, the entropy generation [47] is always non-negative. Final material failure occurs when the entropy inside the material reaches a critical value. Thus, the entropy generation of solid materials under cyclic loading can be used to reveal the degradation of properties and estimate the fatigue lifetime and residual strength of structures. It is worth noting that Naderi et al. [48] have conducted a series of fatigue tests to examine the validity of the damage criterion based on the entropy. Results showed that the cumulative entropy generation was constant at the time of final failure and was independent of geometrical shape, stress level and frequency. At the instance of final failure, the total entropy generation was defined as the fatigue fracture entropy.

To the best knowledge of authors, the applications of an entropy-based failure criterion to estimate the residual strength of CFRPs are still limited [49,50]. Koyanagi et al. [51,52,53] recently developed a computational framework with an entropy-based failure criterion to study the failure mechanism of a visco-elastic matrix and CFRP cross-ply laminates. It was found that the entropy-based failure criterion can reproduce the effect of load frequency on fatigue failure.

However, the estimation of fatigue lifetimes of CFRPs under randomly loading sequences using the entropy-based failure criterion has not been reported. In this study, the entropy-based failure criterion is proposed to estimate the fatigue lifetime of CFRPs subjected to multi-amplitude cyclic loadings. The organization of the paper is as follows: the entropy-based failure criterion is presented in Section 2; numerical simulations and discussions are presented in Section 3 and the conclusions are drawn in Section 4.

## 2. Numerical Method

### 2.1. Visco-Elastic Constitutive Law

**Figure 1:**
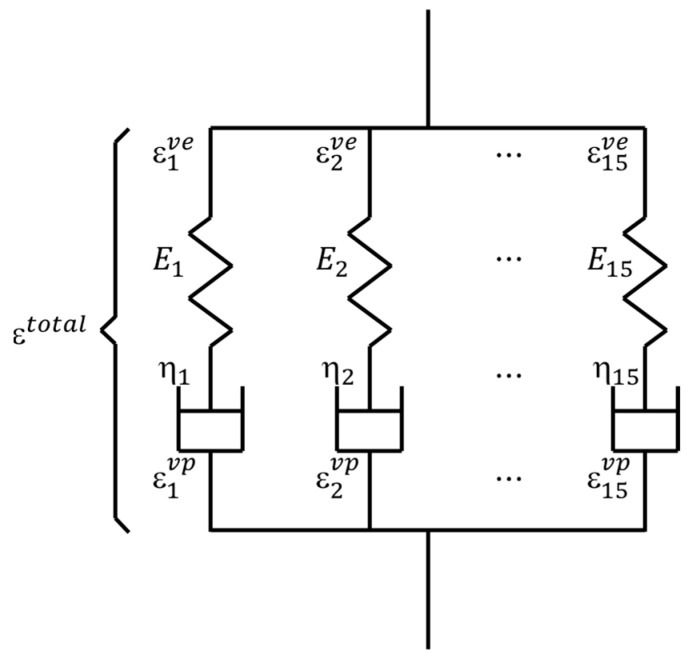
Visco-elastic model with 15 Maxwell elements.

Due to its complexity, the deformation and fracture behaviors of CFRPs are found to be much more complicated than homogeneous materials. In particular, the matrix of CFRPs consists of polymer resin whose properties exhibit high time-dependency. In this study, the visco-elastic constitutive law, based on Maxwell elements, is utilized to characterize the viscosity of the matrix resin, where the number of Maxwell elements is 15 [52], as shown in Figure 1.

In this constitutive law, the total strain ε consists of a visco-elastic strain $\varepsilon^{ve}$ and a visco-plastic strain $\varepsilon^{vp}$ [12]:

(1) $$\varepsilon =\varepsilon^{ve}+\varepsilon^{vp}$$

In Equation (1), the visco-plastic strain $\varepsilon^{vp}$ depends on viscosity and is calculated as

(2) $$\varepsilon^{vp}=\int_{0}^{t}H^{-1}\sigma dt$$

where

(3) $$H=\frac{\eta^{vp}}{\left(1+v\right)\left(1-2v\right)}M$$

(4) $$M=\left[\begin{array}{cccccc} 1-v & v & v & 0 & 0 & 0\\ & 1-v & v & 0 & 0 & 0\\ & & 1-v & 0 & 0 & 0\\ & & & \frac{1}{2}-v & 0 & 0\\ & Symmetry & & & \frac{1}{2}-v & 0\\ & & & & & \frac{1}{2}-v \end{array}\right]$$

(5) $$\eta^{vp}=\eta_{0}\times \frac{1+e^{{\beta \left(\varepsilon_{eqv}^{vp}/\sigma_{eqv}\right)}^{\chi }}}{1+e^{\alpha \left(\sigma_{eqv}-\sigma_{vp0}\right)}}$$

In Equations (3)–(5), *v* is Poisson’s ratio, $\eta_{0}$ is the initial viscosity, α, β and χ are coefficients, $\sigma_{eqv}=\sqrt{\frac{3}{2}s∶s}$ and $\varepsilon_{eqv}^{vp}=\sqrt{\frac{2}{3}\varepsilon^{vp}∶\varepsilon^{vp}}$ are equivalent stress and visco-plastic strain and $\sigma_{vp0}$ is specific stress.

One visco-elastic constitutive law considering the damage D is expressed as [54]

(6) $$\sigma \left(t\right)=(1-D)\int_{0}^{t}E^{r}\left(t-t^{\prime }\right)g{\dot{\varepsilon }}^{ve}dt^{\prime }$$

where the relaxation modulus $E^{r}$ is

(7) $$E^{r}\left(t\right)=\sum_{n=1}^{15}E_{ijkl}^{n}e^{-tE^{n}/\eta^{n}}$$

And the nonlinear coefficient is

(8) $$g=\frac{1}{1+\alpha {\left(\frac{\sigma_{eqv}}{\sigma_{0}}\right)}^{m}}$$

### 2.2. Entropy-Based Failure Criterion

Based on the second law of thermodynamics (the Clausius–Duhem inequality) [48], the irreversible entropy generation in the material is calculated as

(9) $$s=\int_{0}^{t}\left(\frac{1}{T}\sigma :{\dot{\varepsilon }}^{vp}-\frac{A\cdot V}{T}-Q\cdot \frac{\nabla T}{T^{2}}\right)dt$$

where the generalized thermodynamic force vector A is conjugate with the generalized thermodynamic internal flow vector V and Q is the heat flux vector. Practically, entropy generation owing to plastic deformation is dominant, and the last two terms in Equation (9) are negligible. Thus, Equation (9) reduces to [48]

(10) $$s=\int_{0}^{t}\frac{1}{T}\sigma :{\dot{\varepsilon }}^{vp}dt$$

Using Equation (10), the final fracture entropy $s^{f}$ per unit volume of materials can be calculated using the constitutive law or experimental method. When the material is subjected to cyclic loading, the damage variable D, associated with entropy generation s, is introduced to characterize the progressive degradation of material and expressed as [52]

(11) $$D=\frac{s}{s^{f}}D_{cr}$$

where $D_{cr}$ is the user-defined critical damage. In this study, the final fracture entropy $s^{f}$ is assumed to be independent of loading conditions, such as stress level and frequency.

### 2.3. Implementation of the Proposed Method

**Figure 2:**
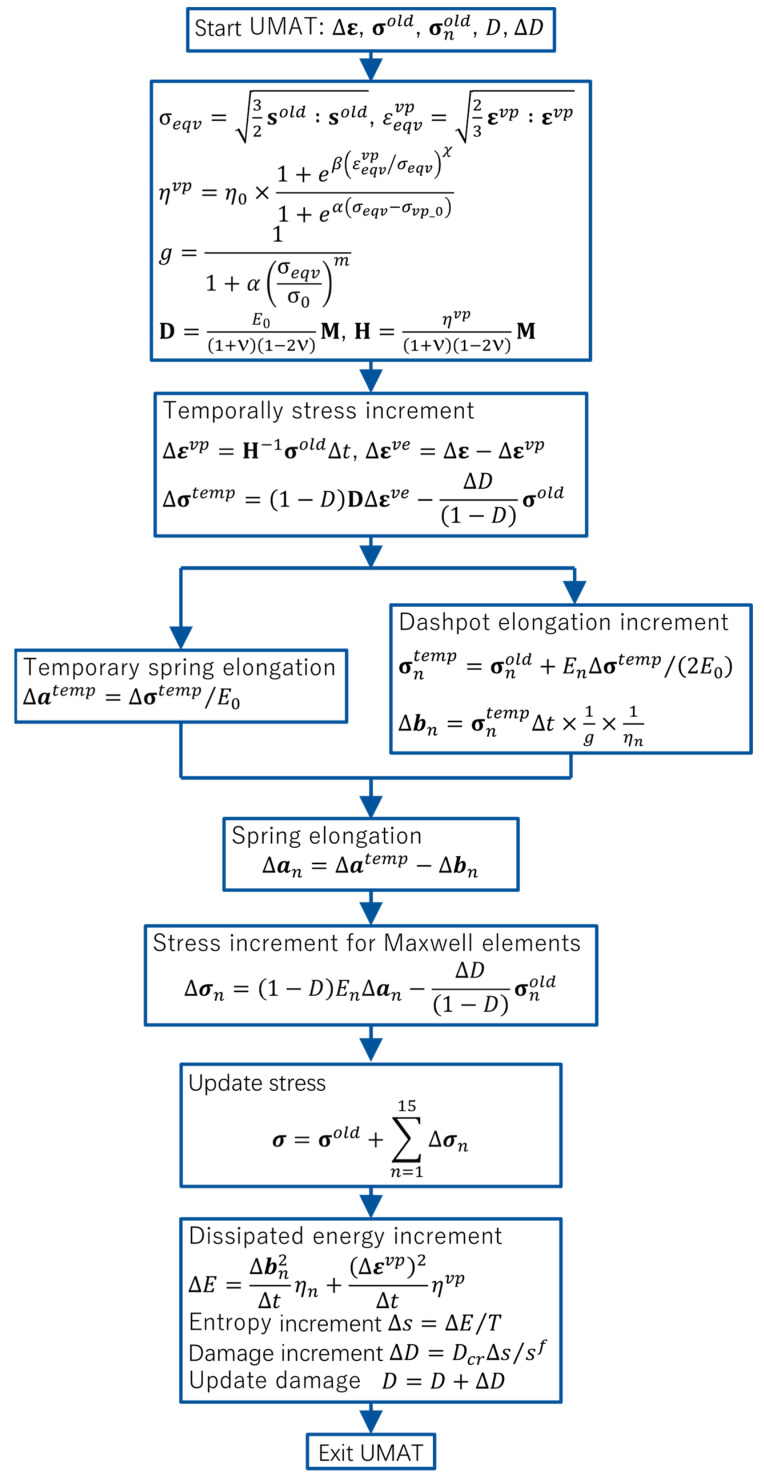
Illustration of updating stress via entropy-based failure criterion.

The proposed entropy-based failure criterion is implemented into a user subroutine to define a material’s mechanical behavior (UMAT) in Abaqus (2020, Dassault Système), and illustrated via a flowchart in Figure 2. With the user subroutine interface, the increments of strain, stress and solution-dependent variables, such as the Maxwell element’s stress $\sigma_{n}^{old}$ and damage D, are passed into UMAT. Based on these, $\sigma_{eqv}$ and $\varepsilon_{eqv}^{vp}$ are firstly computed to determine viscosity $\eta^{vp}$, nonlinear coefficient g and matrices D and H. Additionally, the visco-plastic strain increment $\Delta \varepsilon^{vp}$ is computed to update the increment of visco-elastic strain $\Delta \varepsilon^{ve}$ and temporal stress increment $∆\sigma^{temp}$. Then, the temporal spring and dashpot elongations are calculated to update the stress increment of the Maxwell elements $∆\sigma_{n}$. Finally, the stress at the integration point σ, dissipated energy increment ∆E and entropy increment ∆s are computed to update the damage D and damage increment ∆D.

## 3. Numerical Results and Discussions

**Figure 3:**
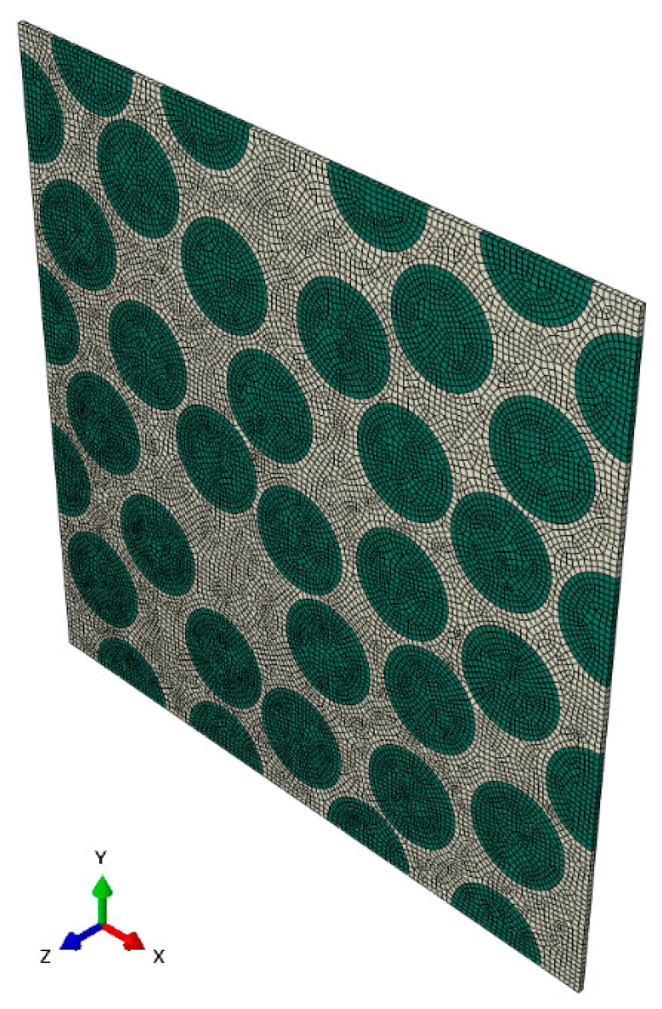
A 3D unit cell model with 37 fibers: green mesh denotes fiber and white is resin.

**Figure 4:**
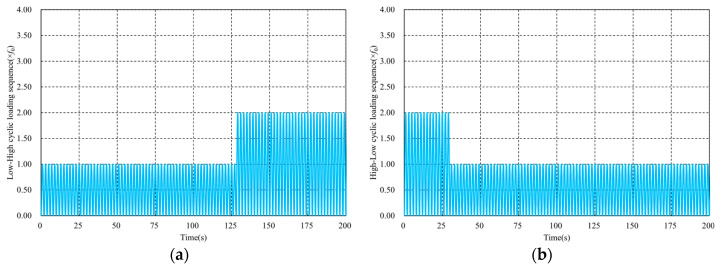
Illustrations of multiple-amplitude cyclic loading sequences. (**a**) Low-high cyclic loading sequence. (**b**) High-low cyclic loading sequence.

In this section, the entropy-based failure criterion is applied to estimate the lifetime of CFRPs under a multiple-amplitude loading sequence. As shown in Figure 3, a three-dimensional (3D) unit cell with dimensions of 39 μm × 39 μm × 0.3 μm [52,55] is utilized to conduct the numerical simulation, in which the diameter of the fibers is 6 μm, the number of fibers is 37 and the volume fraction is 56%. The discretization scheme of this model consists of 36,718 nodes and 18,122 C3D8 elements. The carbon fiber is assumed to be an orthotropic elastic material and its properties are listed in Table 1. The matrix resin is modeled as a visco-elastic medium whose constitutive law is implemented using a user-subroutine UMAT of Abaqus, and its properties are listed in Table 2. A larger $\alpha_{d}$ is chosen to accelerate the evolution of damage and to reduce the simulation time. The displacement boundary condition and external force are employed via the method in the references [52,55,56,57]. In the case of multiple-amplitude cyclic loadings, the loading sequence only consists of two stages, illustrated in Figure 4, both of which are considered in conducting the numerical simulation, in which the lower cyclic loading sequence is expressed as $f_{low}=f_{0}\left[\frac{1}{2}sin\left(\pi t+\frac{3}{2}\pi \right)+\frac{1}{2}\right]$, and the higher sequence is $f_{high}=2f_{low}$, where $f_{0}=1.17\times 10^{-4}$.

### 3.1. Fatigue Lifetime under Multiple-Amplitude Loading

**Figure 5:**
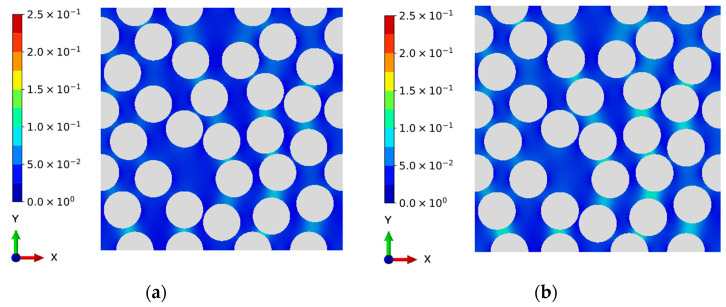

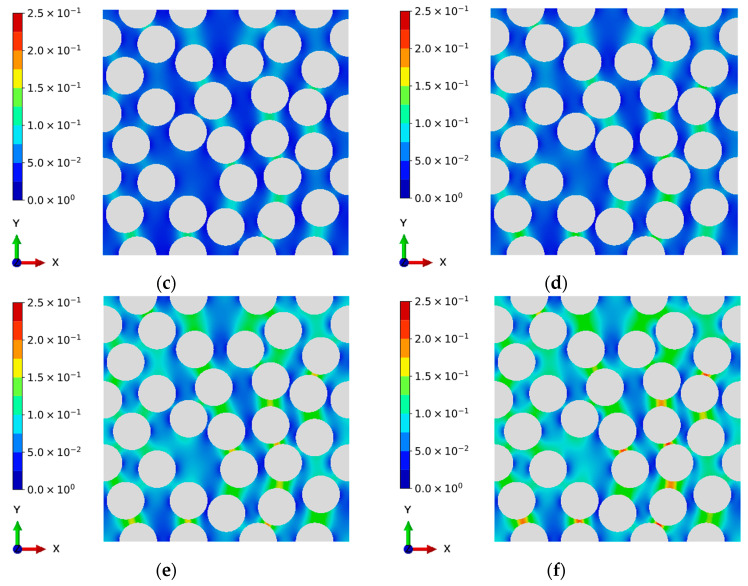
Evolution of damage in CFRPs under low-high cyclic loading sequence. (Palette represents the damage). (**a**) Low stress level: 30 cyclic loadings. (**b**) Low stress level: 40 cyclic loadings. (**c**) Low stress level: 50 cyclic loadings. (**d**) Low stress level: 60 cyclic loadings. (**e**) High stress level: 10 cyclic loadings. (**f**) High stress level: 15 cyclic loadings.

**Figure 6:**
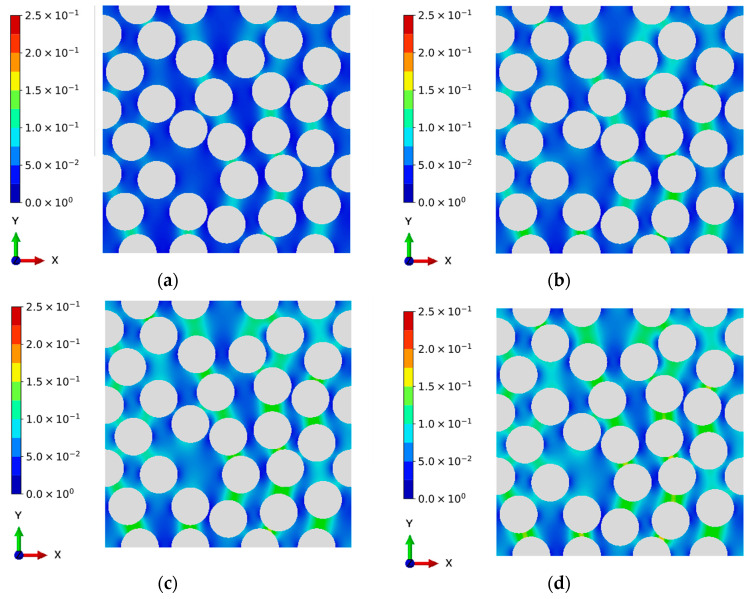

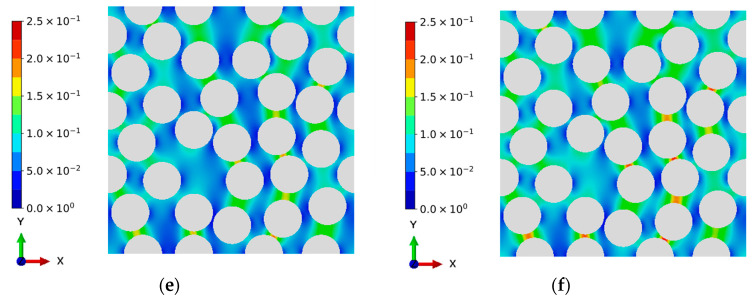
Evolution of damage in CFRPs under high-low cyclic loading sequence. (Palette represents the damage). (**a**) High stress level: 10 cyclic loadings. (**b**) High stress level: 15 cyclic loadings. (**c**) Low stress level: 20 cyclic loadings. (**d**) Low stress level: 30 cyclic loadings. (**e**) Low stress level: 40 cyclic loadings. (**f**) Low stress level: 59 cyclic loadings.

Figure 5 shows the damage evolution of CFRPs under cyclic L-H loading sequences, in which the number of cyclic loadings of the lower level is 64. It can be found that the damage generation initially occurs around the region where two fibers are close to each other. This can be explained by the fact that the deformation variables, such as strain and displacement fields, near this region exhibit significant discontinuity due to the material mismatch between the matrix resin and fibers. In this study, the matrix resin is assumed to be fully failed when the damage variable increases to 0.25 [46], after which the stress will be a very low value, and the initial micro-cracks will be formed between the fiber and the matrix. Additionally, the damage evolutions of the H-L case, where the number of first state sequences is 15, are shown in Figure 6. Analogous to the observations of the L-H loading sequence, the damage accumulations can also be found near the critical region. This phenomenon of damage evolution can also be found in the works of Sato et al. [52], in which only monotonic loading was addressed. Differing from the results of Sato et al. [52], the evolution of accumulated damage caused by the generation of entropy is originally simulated in the current study. As the number of cyclic loadings increases, the damage accumulation is more obvious due to the entropy generation caused by the visco-elastic property of the matrix resin. Thus, the proposed entropy-based failure criterion can reveal the fatigue damage evolution qualitatively.

**Figure 7:**
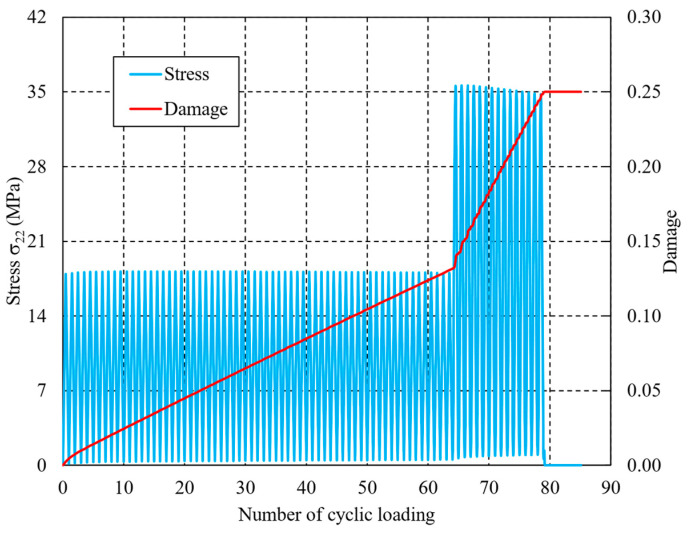
Variations in stress and damage of the element from the initial loading to full failure under the low-high cyclic loading sequence.

**Figure 8:**
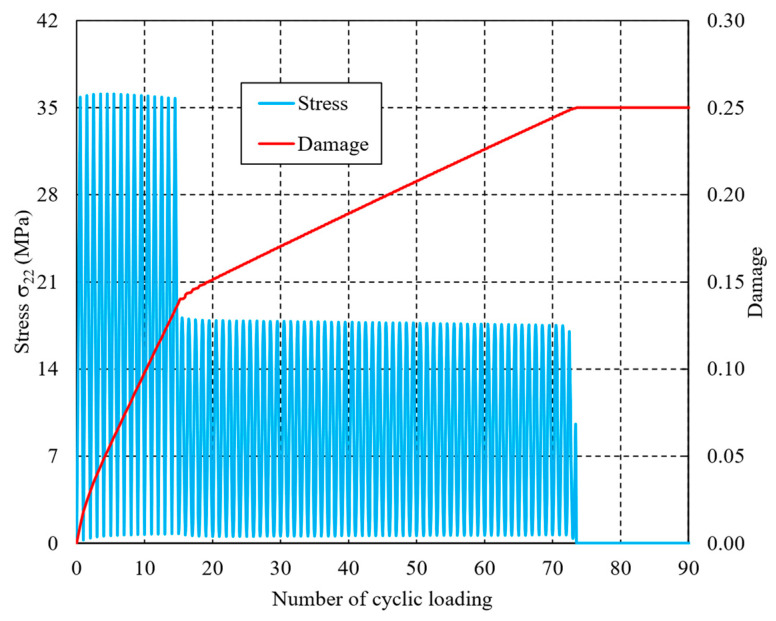
Variations in stress and damage of the element from the initial loading to full failure under the high-low cyclic loading sequence.

Figure 7 and Figure 8 show the variations in stress $\sigma_{22}$ and damage variable D in the earliest element to completely fail. From the initial stage, the dashpots of the Maxwell element move individually due to the cyclic loading, and the dissipated energy gradually accumulates at the same time. The entropy generation can be determined by dividing the dissipated energy by temperature to update the damage variable and degrade the material properties. The higher the loading sequence, the faster the entropy or damage variable increases. Thus, the effect of the loading sequence on the fatigue lifetime of CFRPs can be revealed. In the L-H loading sequence, the damage variable increases to 0.1316 after 64 lower cyclic loadings, after which it reaches 0.25 with 15 higher cyclic loadings. Thus, the total lifetime to full failure can be determined as 64 + 15 = 79. However, in the H-L case, i.e., when the order of lower and higher loading sequences are exchanged, where the number of cyclic loadings in the higher level is taken as 15 (that is, the same as the value in the case of L-H), the damage variable reaches 0.1380 after the first higher level, and 0.25 after 59 lower cyclic loadings. Thus, the predicted total lifetime to final failure in the H-L case is 15 + 59 = 74.

By comparing the predicted fatigue lifetimes of the two cases, it can be observed that a significant difference of values, (79 − 74)/79 = 6.3%, occurs. This difference can be utilized to illustrate the dependence of loading sequence on the fatigue lifetimes of components subjected to random loadings [19,20,21]. To the best knowledge of the authors, the proposed entropy-based failure criterion is the first that has the ability to reproduce the effects of loading sequence on the fatigue lifetime. As the number of cyclic loadings increases, the fatigue lifetime of the H-L loading sequence will be reduced more significantly than the L-H lifetime. It is worth noting that the proposed methodology requires a higher computational cost due to the determination of dashpot elongation in the Maxwell elements. In particular, the computational costs will increase dramatically in the case of cross-ply laminates. In addition to the conventional material properties, the final fracture entropy $s^{f}$ should also be determined experimentally [52] when dealing with various matrix resins.

### 3.2. Further Discussions

Before applying the entropy-based failure criterion to estimate the fatigue lifetime under multiple-amplitude cyclic loadings, its validity has been reported by Sato et al. [52], in which only monotonic loading was addressed, and comparisons with experimental results are also conducted. In addition, although the micro-scale model is proposed in this study, it is also appropriate to extend the entropy-based failure criterion to the macro-fatigue failure of CFRPs under cyclic loadings. In fact, Koyanagi et al. [51,53] have adopted the entropy-based failure criterion to investigate the macro-transverse crack accumulation behavior of CFRPs under cyclic loadings. In contrast to the micro-scale modeling, the CFRP is assumed to be a homogeneous orthotropic medium, and only five Maxwell elements are utilized to address the visco-elasticity of the CFRP due to the high computational cost. The well-known Hashin criteria are introduced to characterize the typical failure modes, and the entropy generation is used for material property degradation. The numerical results demonstrate that the entropy-based failure criterion can reveal the effect of the load frequency on the transverse crack accumulation behavior. Thus, the proposed failure criterion is a promising method to predict the residual strengths and fatigue lifetimes of CFRP components.

### 3.3. Comparisons with LCD and KCG Methodologies

In this section, the fatigue lifetime of the matrix resin is estimated via the conventional linear cumulative damage theory and the kinetic crack growth theory. The failure criterion of LCD [58] is expressed as

(12) $$\int_{0}^{\tilde{t}}\frac{{\tilde{\sigma }}^{1/n+1}\left(\tau \right)d\tau }{1-{\tilde{\sigma }}^{1/n}\left(\tau \right)}=1$$

And the failure criterion of KCG [44] is

(13) $$\frac{1}{1-{\tilde{\sigma }\left(\tilde{t}\right)}^{1/n}}\int_{0}^{\tilde{t}}{\tilde{\sigma }}^{\left(1/n+1\right)}\left(\tau \right)d\tau =1$$

where $\tilde{\sigma }$ and $\tilde{t}$ are the normalized stress and time and the material property parameter is taken as n=1.0703.

**Figure 9:**
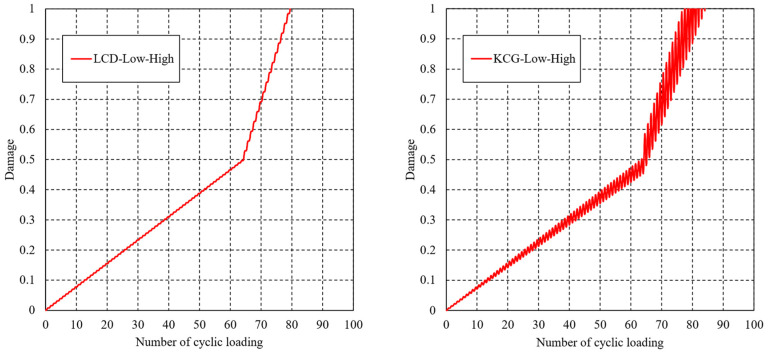
Variations in damage determined by LCD and KCG theories in the L-H loading sequence.

**Figure 10:**
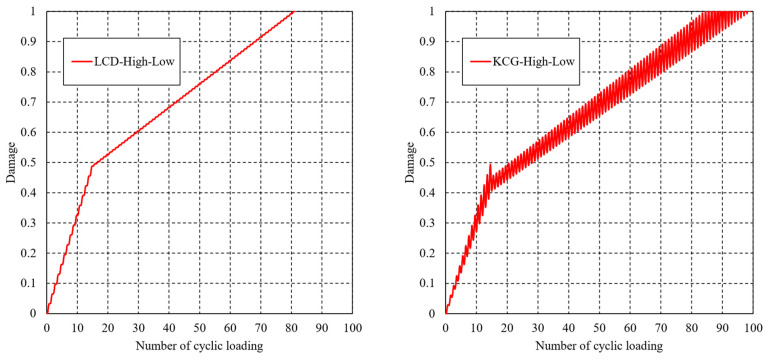
Variations in damage determined by LCD and KCG theories in the H-L loading sequence.

Figure 9 compares the variation in damage determined by Equations (12) and (13) versus the number of cyclic loadings in the case of the L-H loading sequence. The damage variable continues increasing after the beginning of cyclic loading, and exhibits differently increasing rates in the various loading levels. It is worth noting that the variation law of the results from LCD is consistent with that from the entropy-based failure criterion. However, significant oscillation phenomenon can be found in the KCG method. The predicted lifetimes of the two methodologies are 80 and 77, respectively, which are in good in agreement with the entropy-based failure criterion’s lifetime of 79. In the case of the H-L loading sequence, the variation in damage is shown in Figure 10, and the estimated lifetimes are 81 and 85, respectively.

However, the estimated lifetimes in the case of the H-L loading sequence are always higher than in L-H, regardless of whether the LCD or KCG methodology is used. This observation is contrary to the conclusion discussed in Section 3.1. Additional studies and experiments, which will be presented soon, need to be introduced for a further explanation.

## 4. Conclusions

The entropy-based failure criterion that can reveal the effect of the loading sequence on the fatigue lifetime is implemented into a UMAT of Abaqus to estimate the fatigue lifetime of unidirectional CFRPs subjected to multiple-amplitude cyclic loadings in this study. Due to the heterogeneity of CFRPs, a micro-finite element model, considering matrix resin and fibers separately, is developed for the numerical simulation. The fatigue lifetimes of CFRPs subjected to L-H and H-L loading sequences are estimated by the proposed method. Numerical results demonstrate that the estimated fatigue lifetime under the L-H loading sequence is higher than that under the H-L loading sequence and the difference in predicted lifetime to final failure is 6.3%. Thus, the proposed method can reveal the effect of the loading sequence on the fatigue lifetime. This is a significant advantage compared to the conventional methods to estimate the residual strengths and lifetimes of CFRP structures. Additionally, comparisons with the LCD and KCG theories are also conducted to illustrate the validity of the proposed method. In future works, the effects of heat generation and thermal conduction on the entropy generation and material property degradation will be included.

## Figures and Tables

**Table 1:** Material properties of fiber.

Properties	$E_{11}$$,\;E_{22}$(GPa)	$E_{33}$(GPa)	$v_{13}$ $,\;v_{23}$	$v_{12}$
Value	294	15	0.02	0.3

**Table 2:** Material properties of matrix resin.

n	$E_{n}(MPa)$	$\eta_{n}(MPa$ ·s)	Elasticity	
1	284	4.5 × 10^2^	$E_{0}(MPa)$	4260
2	284	3.3 × 10^3^	*v*	0.3
3	284	1.2 × 10^5^	Nonlinearity	
4	284	1.9 × 10^6^	$\sigma_{0}(MPa)$	70
5	284	1.8 × 10^7^	α	2
6	284	1.4 × 10^8^	*m*	7
7	284	8.5 × 10^8^	Visco-plastic strain	
8	284	5.0 × 10^9^	$\eta_{0}(MPa\cdot s)$	1.0 × 10^23^
9	284	3.0 × 10^10^	$\sigma_{vp\_0}(MPa)$	0
10	284	1.9 × 10^11^	$\alpha_{vp}$	0
11	284	1.4 × 10^16^	$\beta_{vp}$	0
12	284	1.3 × 10^19^	*χ*	0
13	284	2.1 × 10^22^		
14	284	1.3 × 10^26^	Damage variables	
15	284	2.5 × 10^29^	$\alpha_{d}$	4

## Data Availability

The data that support the findings of this study are available from the corresponding author upon reasonable request.

## Nomenclature

ε, Δε: Total strain tensor, increment of total strain tensor.
$\varepsilon^{ve}$, $\varepsilon^{vp}$: Visco-elasticity strain tensor, visco-plasticity strain tensor.
σ, Δσ, s: Stress tensor, increment of stress tensor, deviatoric stress tensor.
p, I: Hydrostatic stress, second order identity tensor.
D, $D_{cr}$: Damage variable, critical damage variable.
s, $s^{f}$: Entropy generation, final fracture entropy.
g: Nonlinear coefficient.
t, $t^{\prime }$, *τ*: Time.
W, $\alpha_{d}$: Dissipated energy, damage parameters.
$E^{r}$, H, M: Relaxation tensor, viscosity matrix, constant matrix.
D, $E_{0}$, *v*: Stiffness matrix, initial Young’s modulus, Poisson’s ratio.
$E_{n}$ $\eta_{n}$: Visco-elasticity properties of Maxwell elements.
$\sigma_{eqv}$, $\varepsilon_{eqv}^{vp}$: Equivalent stress, equivalent visco-plastic strain.
$\eta^{vp}$, $\eta_{0}$, *α*, *β*, χ: Visco-plasticity coefficients.
A, V: Generalized thermodynamic force and internal flow vectors.
T, Q: Temperature, heat flux vector.
